# nVenn2: faster, simpler generalized quasi-proportional Venn diagrams

**DOI:** 10.1093/bioadv/vbag183

**Published:** 2026-06-27

**Authors:** Samuel Pis-Vigil, María González-Pereira, Magda R Hamczyk, Víctor Quesada

**Affiliations:** Departamento de Bioquímica y Biología Molecular, Universidad de Oviedo-IUOPA, Asturias 33006, Spain; Departamento de Bioquímica y Biología Molecular, Universidad de Oviedo-IUOPA, Asturias 33006, Spain; Aarhus Institute of Advanced Studies, Aarhus University, Aarhus DK-8000, Denmark; Departamento de Bioquímica y Biología Molecular, Universidad de Oviedo-IUOPA, Asturias 33006, Spain; Instituto de Investigación Sanitaria del Principado de Asturias (ISPA), Oviedo 33011, Spain; Centro de Investigación Biomédica en Red de Cáncer (Ciberonc), Instituto de Salud Carlos III, Madrid 28029, Spain

## Abstract

*Motivation:* Proportional Venn diagrams provide a compact representation of the relationships between sets. Each relationship is represented with a region whose area reflects the number of elements shared by a given combination of sets. This means that the number of regions grows exponentially with the number of sets, which is why proportional Venn diagrams with more than five sets are cumbersome to interpret and seldom used. However, Venn diagrams with a large number of sets may still be legible if enough regions are empty and do not need to be represented.

*Results:* Here, we present nVenn2, the second version of the nVenn algorithm, to create quasi-proportional Venn diagrams. This new version uses a different, more flexible approach which includes steps to minimize the complexity of the diagram. Thus, computation time for nVenn2 mainly grows with the number of non-empty diagram regions, rather than with the number of sets. This property allows users to create interpretable quasi-proportional Venn diagrams with large numbers of sets.

*Availability and implementation:* The nVenn2 algorithm is freely available as an executable program, as a web page, as an R package (nVennR2) and as a Python package (nVennPy). All interfaces allow users to edit the appearance of the resulting diagram.

## 1 Introduction

Venn diagrams were first described in the 19th century as tools for logical reasoning (Venn 1880). John Venn started with ideas from Leonhard Euler and others, in combination with the logical system developed by George Boole, to represent complex propositions in a single figure. Venn himself noticed that “[b]eyond five terms it hardly seems as if diagrams offered much substantial help,” since the number of propositions doubles with each new term. It is interesting to notice that this original description is focused on how negative propositions can be represented by eliminating regions in the general diagram. As the original aim of this representation was to exhaust the logical propositions from a number of terms, eliminating regions in a diagram was never considered as a strategy to make it more readable.

As a trivial extension of their use in the field of logic, Venn diagrams have also been used to illustrate basic concepts of set theory, such as “union” and “intersection” (Ashlock and Lee 2020). This use emphasizes the contents of sets as a measure of how similar or dissimilar those sets are. In this context, it makes more sense to eliminate regions corresponding to inexistent relationships between sets. For instance, if two sets have no elements in common, we can erase their intersection and depict them as two disjoint circles. Some authors refer to Venn diagrams with empty regions as Euler diagrams, even though Venn diagrams are historically a refinement of Euler diagrams (Moktefi *et al.* 2012).

Moreover, a Venn diagram can be drawn in such a way that the area of each region is proportional to the number of elements in that region. This representation provides a quick overview of the relationships between sets, expressed as the proportion of elements they share. In bioinformatics, obtaining this overview is a recurrent task. A typical use involves sets of genes that are identified in different conditions of a given experiment. The proportional Venn diagram of those sets allows researchers to quickly detect abnormal regions (e.g. “most of the genes identified in condition A are also identified in condition B, but not in C”) and to obtain a list of the elements in each region (e.g. “which genes were identified in conditions A and B, but not in C?”). Certain sub-fields, like transcriptomics and proteomics, make extensive use of Venn diagrams to quickly compare conditions when hundreds or thousands of elements are measured per experiment.

Representing proportional Venn diagrams with more than two sets is challenging, and cannot be done in the general case using convex shapes. However, multiple programs attempt to solve this task in some limited form (Jia *et al.* 2021), consistent with the importance of these diagrams in bioinformatics and other fields. Some of these programs, like eulerAPE (Micallef and Rodgers 2014b), use regular shapes and limit the number of sets that may be represented. Others, like eulerr (Larsson 2024) and Edeap (Wybrow *et al.* 2021), use ellipses for sets and relax the proportionality constraint by using penalty functions. Other representations focus on optimizing and simplifying the topological properties of the diagram (Rottmann *et al.* 2023, Yan *et al.* 2023).

Our own solution for proportional Venn diagrams, called nVenn, uses irregular shapes, which allow for more accurate diagrams without limiting the number of sets (Pérez-Silva *et al.* 2018). Thus, the first nVenn algorithm started with a general Venn diagram. Each region of the diagram contained a circle with an area that was proportional to the number of elements in that region. This starting representation was then simplified using a simulation that maintained its topological properties while adjusting the set lines to the circles. The resulting diagrams can be quite convoluted, but, in theory, they can faithfully represent any arbitrary Venn diagram with any number of sets. This characteristic set nVenn apart from other existing algorithms. While nVenn figures were often useful, several limitations existed that were intrinsic to the algorithm.

First, the starting diagram was always identical, and therefore the final figure was always the same for a given input. Since some representations could be sub-optimal (i.e. unnecessarily complex), some diagrams could not be improved without changing the order of the sets, and even in that case an optimal figure might not be obtained. Second, the starting diagram did not take advantage of empty regions, which made the simulation time scale non-linearly with the number of sets. This meant that nVenn diagrams with more than seven sets were often impractical. While in most cases such a diagram would not be useful, there are exceptions, as the interpretability of a proportional Venn diagram depends on the number of non-empty regions rather than on the number of sets.

Here, we describe the second version of the nVenn algorithm, which features a heavily modified procedure to address previous limitations. Thus, nVenn2 gives a different result, with minimized local complexity, each time it is run.

## 2 Implementation

Like the previous version, nVenn2 is coded in C++. To simplify the process of connecting different interfaces, new functions have been added to the core C++ libraries. The input of nVenn2 is a table where each row or column, as decided by the user, provides a set name and set elements. The corresponding description of the Venn diagram is then computed. In addition to running the simulation to get the diagram, the core libraries also allow users to explore the diagram by listing the elements corresponding to each region. An additional set of new functions can be used to edit the resulting diagram by changing colors, font sizes, and other graphical features.

### 2.1 Steps

**Figure 1:**
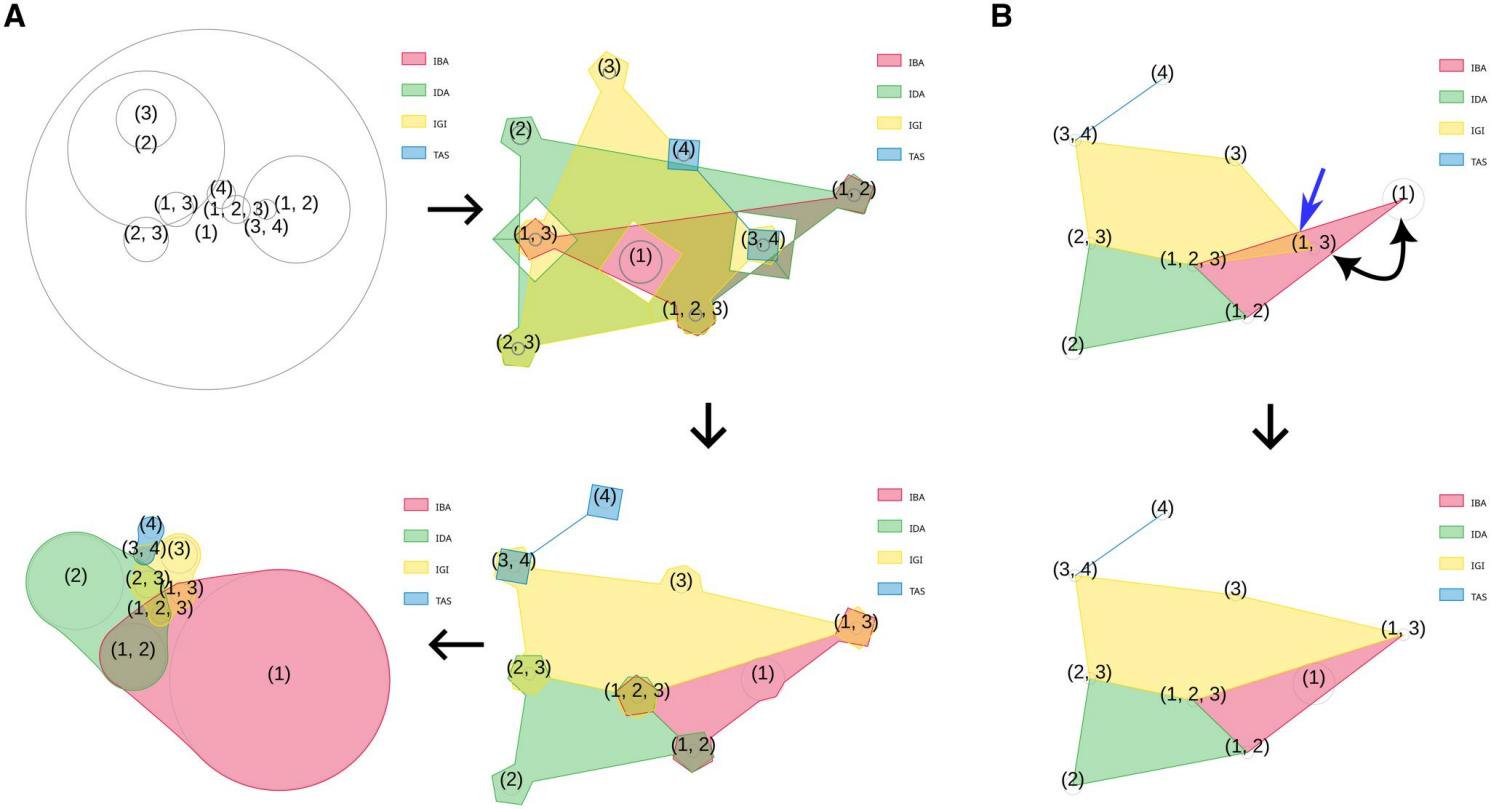
nVenn2 algorithm. (A) Circles representing regions are placed on grid pseudorandomly and find an equilibrium where similar regions are attracted and dissimilar regions are repulsed (upper left). A repulsive force separates circles into a new grid and a topologically correct Venn diagram is generated with lines surrounding all regions (upper right). Then, regions are swapped pairwise to maximize a measure of how close similar regions are, then to minimize the number of line crossings in the corresponding Venn diagram (lower right). Finally, the lines in the Venn diagram contract until regions are in contact and lines are smoothed (lower left). (B) The last minimization cycle swaps regions (1) and (1,3) (curved arrow) and eliminates a line cross (blue arrow). The universe for these figures contains genes included in the innate immune system GO category (GO: 0045087). The diagram shows four subsets containing the genes with different GO evidence codes: Inferred from Biological aspect of Ancestor (IBA), Inferred from Direct Assay (IDA), Inferred from Genetic Interaction (IGI), Traceable Author Statement (TAS). Number lists in parentheses show the sets each region belongs to, in the same order as in the legend.

The final diagram is generated in seven steps (Fig. 1 and Supplementary Video, available as supplementary data at *Bioinformatics Advances* online). Each of these steps is finished when specific criteria have been met. This ensures that the simulation is not run for more steps than necessary.

In the first step (Fig. 1A, upper left), each region is represented as a circle whose radius is proportional to the square root of the number of elements in the region, so that its area is proportional to that number. The starting coordinates for each circle are set pseudorandomly at pre-defined distances from the origin. Therefore, for a diagram with $n_{r}$ regions, the number of starting combinations is at least $n_{r}!$. In practice, this number is usually larger, as these pre-defined positions can remain empty.

Then, a simulation is run with a dissipative friction force (of the type −bv, where *b* is a constant factor and *v* is the speed of the circle) and a spring-like force between the centers of each pair of circles (of the type −kΔr, where *k* is a constant factor that may be negative for repulsion and Δr is the distance between the point centers). This spring-like force is stronger the more sets the corresponding regions have in common, and it becomes repulsive if the corresponding regions do not both belong to any common set. For instance, a circle (i.e. region) that belongs to sets 1, 2, and 4 will be attracted to the circle that belongs to sets 1 and 4 more strongly than to the region that belongs only to set 1, while it will be repulsed by the circle that belongs only to set 3. Once the total velocity of all circles is lower than a certain threshold, the second step sets a repulsive force between all the circles. The simulation is run until the minimum distance between circles is higher than a preset threshold. At this point, a dedicated function adds set lines that include every region belonging to each set and possibly other regions. A second function modifies those set lines to exclude regions that do not belong to each set (Fig. 1A, upper right, Supplementary Data and Fig. S1, available as supplementary data at *Bioinformatics Advances* online).

The two following steps minimize some property of the resulting grid. First, the value of the property is calculated. Then, the coordinates of two circles are swapped, the set lines are re-formed and the property is calculated again. If the new value is higher than the previous value, the swap is undone. Each step is repeated for every pair of regions until a certain number of steps (equal to the number of regions in the diagram) has not improved the value of the main property (Fig. 1A, lower right).

Thus, the third step attempts to minimize a property we have called compactness (Supplementary Methods, available as supplementary data at *Bioinformatics Advances* online), based on the product of the distances between regions belonging to the same set. The value of this property is lower when regions belonging to the same are closer together. At the end of this stage, set lines will be more compact, but the overall complexity of the figure is still sub-optimal. The fourth step uses the same strategy to minimize the number of line crossings between sets (Fig. 1B). The rationale of this step is based on the observation that unnecessarily complex Venn diagrams contain more set crossings than simpler figures.

This minimization procedure does not explore every possible region swap, only favorable swaps from the starting diagram. To explore the combinatorial space in a systematic way, the algorithm can be run in depth-level-wise exhaustive mode. At the first level, every region pair is swapped and the algorithm keeps the diagram with the lowest number of line crossings. With two levels, the algorithm also explores every possible two-pair swap. Increasing this depth level close to the number of regions ensures that the final diagram has the lowest number of crossings possible, but this requires considering $O(n^{n})$ combinations on the number of regions. The procedure also blocks the thread where it is running. For these reasons, a function is provided to estimate the time the algorithm will need to complete the minimization step in exhaustive mode at a given level.

Once the minimization steps are completed, three additional steps simulate a physical system to reach a compact diagram (Fig. 1A, lower left). These steps are similar to the simulation steps in the first version of nVenn (Pérez-Silva *et al.* 2018) with some improvements in speed and stability. The most important change is that after each step a diagram property (compactness or area) is calculated and compared to that of previous steps. If this property is not consistently improving, the step is finished.

Briefly, the fifth step simulates attracting spring forces between the set border points. Regions are kept non-overlapping by adding contact forces. The step is finished when compactness is no longer improving. The sixth step keeps those forces and adds a gravity-like force (of the type $-G/\Delta r^{2}$) between regions to further compact the figure. This step is also finished when compactness is no longer improving. Finally in the seventh step the coordinates for the regions are kept fixed and additional points are added to the set borders. a spring-like attractive force is added between those points and a gravity-like force is added between set border points and regions. The simulation is ended when the total area enclosed by the set lines is no longer decreasing. More details about each step can be found in Supplementary Methods, available as supplementary data at *Bioinformatics Advances* online.

### 2.2 Interfaces

In addition to direct compilation, nVenn2 can be used from a web page, R, and Python. Each of these interfaces uses the C++ files without modifications, so that improvements to the libraries can be easily propagated.

#### 2.2.1 Web page

The web page uses HTML, CSS, vanilla javascript, and SVG for the interface. The library itself was compiled into WebAssembly (Haas *et al.* 2017) using Emscripten v3.1.69 (https://emscripten.org/). The text table describing each set can be directly pasted into a text area or read from a file.

Once the diagram is generated, the user can explore it using tick boxes or directly clicking on regions. A text box will show the elements exclusively belonging to the provided region. A new version of the diagram can be created by simply clicking a button.

The interface contains several controls to rotate and modify the resulting figures. A diagram can be saved as an SVG file or standalone web page, or exported as a PNG (bitmap) file. Saved SVG or web page files can be reloaded into the interface for further processing. A tutorial provides step-by-step instructions to create, explore, edit and store diagrams.

#### 2.2.2 R library

The R interface uses Rcpp (Eddelbuettel and Balamuta 2018) to create function bindings to the library methods. Since a C++ object is not trivial to store in R, only equivalent R objects are saved. When those R objects are passed to Rcpp methods, the C++ library can read the code and internally rebuild the original C++ object. In keeping with the functional paradigm of R, all methods return a modified R object without changing the original object.

An advantage of Rcpp is that R types can be easily passed to C++ code. Therefore, this interface allows users to create an object from a text table and also from a list of lists, similar to the first version of nVennR. A vignette is provided with examples of use.

#### 2.2.3 Python library

The Python library contains bindings created with Pybind11 (https://github.com/pybind/pybind11). This interface uses the bindings directly to create a Python object that can be accessed and modified. The diagram contained in the object can be edited, saved, and exported. This version only creates objects from text tables.

An interactive, publicly available Jupyter Notebook in Binder documents the functions with examples (https://mybinder.org/v2/gh/vqf/codespaces-jupyter/HEAD?urlpath=%2Fdoc%2Ftree%2Fnotebooks%2Fdoc.ipynb).

## 3 Testing

To compare the first and second versions of nVenn, we generated pseudorandom Venn diagrams in R and represented them using nVennR and nVennR2. For each experiment, a universe of predefined size was populated with sequential integers and sets were generated by randomly sampling elements from that universe. Three universe sizes (20, 30, and 50 elements) and different numbers of sets were evaluated. For each combination of universe size and number of sets, 40 independent diagrams were generated. Execution times were recorded and the number of non-empty regions in each resulting diagram was stored for subsequent analyses. Since nVennR does not include an automatic convergence criterion, diagrams were refined through repeated 5000-cycle optimization blocks. The number of refinement blocks was empirically adjusted according to the number of sets to obtain consistently compact diagrams. For nVennR2, execution time corresponded to a single complete run of the algorithm, whereas for nVennR the total time included all refinement iterations required for convergence. All scripts used for benchmarking are available at https://github.com/uo283276/Pruebas_nVenn.

**Figure 2:**
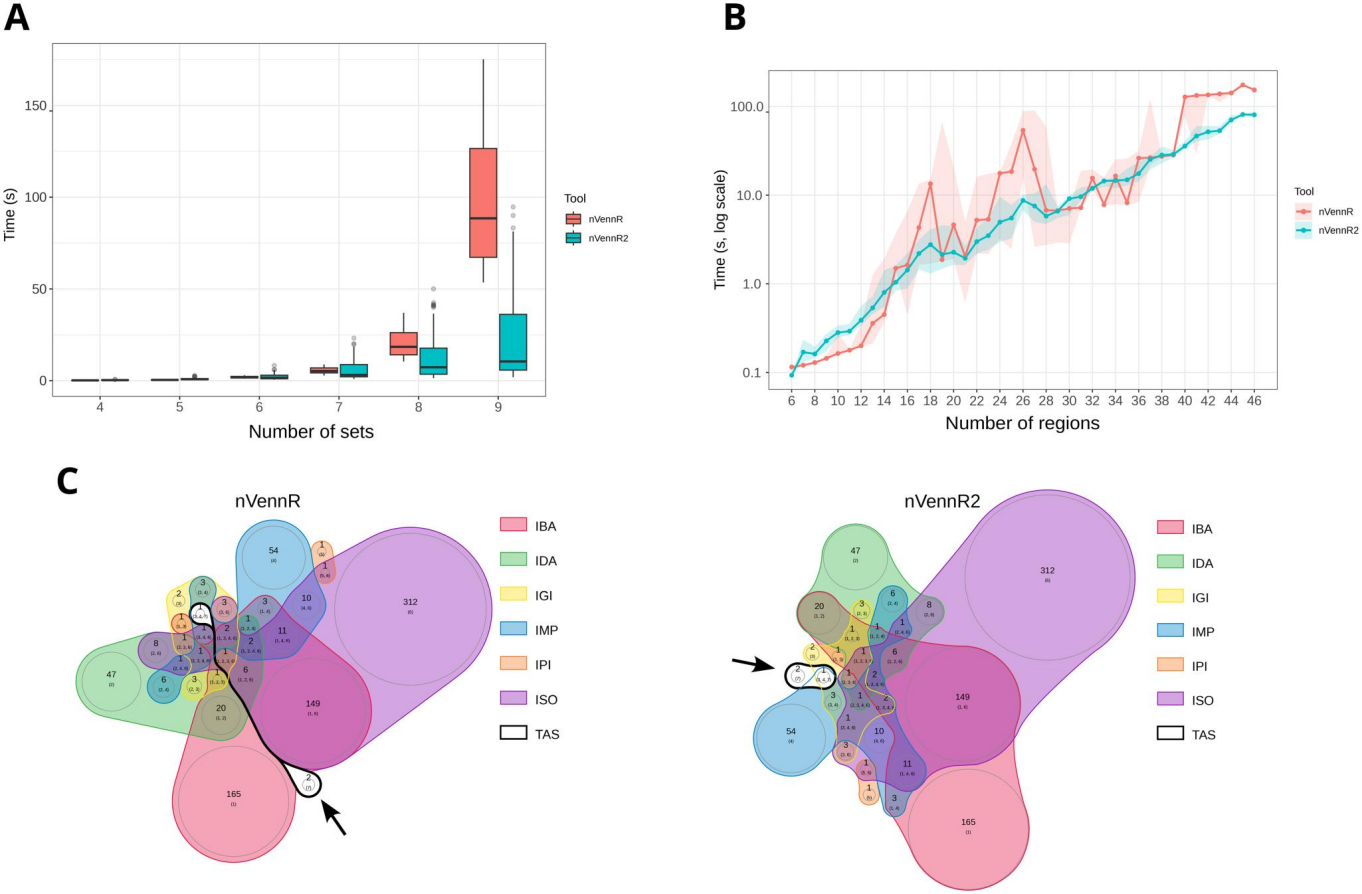
Performance of nVenn algorithms. (A) Execution time for diagram completion with different numbers of sets. Boxes show the span from the first and third quartiles of the results, black horizontal lines show the median and whiskers represent an ∼95% confidence interval (*N* = 40 for each number of sets). (B) Execution time for diagram completion with different numbers of regions. Dots show the median time for completion and shaded areas represent an ∼95% confidence interval (*N* = 40 for each number of regions). (C) Seven-set diagram constructed with nVennR (left) and nVennR2 (right). The universe for the figures contains genes included in the innate immune system GO category (GO: 0045087). Seven subsets with different GO evidence codes were generated: Inferred from Biological aspect of Ancestor (IBA), Inferred from Direct Assay (IDA), Inferred from Genetic Interaction (IGI), Inferred from Mutant Phenotype (IMP), Inferred from Physical Interaction (IPI), Inferred from Sequence Orthology (ISO), and Traceable Author Statement (TAS). Arrows point to set 7 (TAS). Number lists in parentheses show the sets each region belongs to, in the same order as in the legend.

As expected, the performance of both algorithms was very similar with diagrams up to five sets (Fig. 2A). For six or more sets, nVennR2 was consistently faster than nVennR. In fact, Venn diagrams with more than nine sets were feasible with nVennR2 and not with nVennR. We also represented the computational time needed relative to the number of regions in the diagram (Fig. 2B). Again, execution times were similar for diagrams with few regions, although nVennR2 showed more consistency between runs. For higher numbers of regions, nVennR2 was clearly faster and much more consistent.

In addition, we found multiple anecdotal examples of nVennR diagrams that were improved by nVennR2. For a comparison, we downloaded all the protein codes for genes included in the innate immune system GO category (GO: 0045087) and prepared a table with seven different GO evidence codes for each of them (Table S1, available as supplementary data at *Bioinformatics Advances* online). The corresponding diagram performed with nVennR shows non-optimal placement of some regions (Fig. 2C). Thus, the relationship of set seven (TAS, Traceable Author Statement) with the rest of sets is much easier to interpret with nVennR2. Namely, out of the three proteins tagged with the TAS tag, two are not tagged with any other evidence code and one is also tagged with both IGI (Inferred from Genetic Interaction) and IMP (Inferred from Mutant Phenotype).

## 4 Discussion

The aim of nVenn2 is to provide a faithful and esthetically pleasing representation of the relationships between sets, as well as a set of tools to explore the corresponding regions. The representation itself follows the same criteria as the first version of the algorithm. Notably, this means that each region is represented by the corresponding circle, whose area is proportional to the size of the region. The set line surrounding those circles does not necessarily follow that proportionality. For this reason, nVenn2 does not provide any mechanism to alter the circles representing regions. Moreover, the radius of any circle may not be <2% of the width of the diagram, which means that the area of smaller regions may not faithfully represent the number of elements it contains. With these caveats, nVenn2 sacrifices regularity of set shapes to become the only tool that faithfully represents general n-dimensional proportional Venn diagrams (Table 1).

**Table 1:** Comparison of Venn diagram generators.

Program	Maximum number of sets	Proportional areas	Fidelity	Symmetric set shapes	Interfaces	Reference
BioVenn	3	Yes^a^	May show empty regions	Yes	Web, R, Python	Hulsen *et al.* (2008)
venneuler	4	Yes^a^	May show empty regions	Yes	R	R package
pyvenn	6	No	May show empty regions	Yes	Python	Python package
jvenn	6	No	May show empty regions	Yes	Web	Bardou *et al.* (2014)
eulerr	Unlimited	Yes^a^	May show empty regions May not show existing regions.	Yes	R	R package
Edeap	Unlimited	Yes^a^	May show empty regions May not show existing regions.	Yes	Web	Wybrow *et al.* (2021)
DeepVenn	Unlimited	Yes^a^	May show empty regions May not show existing regions.	Yes	Web	Hulsen (2022)
vennmaster	Unlimited	Yes^a^	May show empty regions May not show existing regions.	Yes	Java app, GoMiner	Kestler *et al.* (2008)
eulerForce	Unlimited	No	May show empty regions May not show existing regions.	No	Java app	Micallef and Rodgers (2014a)
nVenn2	Unlimited	Yes^b^	All existing regions shown No empty regions shown.	No	Web, R, Python	Present manuscript

a Not strict, using loss function.

b Small regions may become larger for readability.

The second version of nVenn features a complete rework of the original algorithm. Only the last three steps of nVenn2 have counterparts in the first version, and they have been streamlined. At the core of the new algorithm, a procedure generates a topologically correct diagram from a grid of circles representing regions. In particularly complex cases, this step can fail. However, we found no errors during testing with hundreds of Venn diagrams, which suggests that the error frequency is exceedingly low.

Another fundamental difference between versions is that nVenn2 yields a different diagram after every run. Users are encouraged to try several simulations to get a figure that best represents the data. With this feature, we have been able to balance exhaustiveness and running time. Thus, the topology of the diagram is decided using a form of gradient descent. While this is usually good enough for simple diagrams, in more complex situations the algorithm will likely stop at a local minimum. A procedure that looks for global minima in arbitrary cases is also provided, but it may require large computational resources and running time. This setting may be convenient when the diagram is expected to be consistently rendered in the best possible representation, and its execution may be made conditional on its estimated running time.

It is also worth noting that the source of randomness in the code is the mt19937 Mersenne twister engine fed by the C++ standard random device. The definition of this random device allows implementations that yield the same number sequence every time. If the code is compiled with this implementation, the same input will yield the same output every time the program is started. In this case, repeated execution without closing the program will still produce different diagrams. The compilers for the provided interfaces do not have this limitation.

In summary, the second version of nVenn overcomes some of the most obvious limitations in the first version. In most cases, nVenn2 will be faster and provide more informative diagrams. In addition, we are providing multiple interfaces to allow for fast integration of nVenn2 with existing workflows.

## Supplementary Material

vbag183_Supplementary_Data

## Data Availability

All libraries and interfaces are publicly available under MIT licenses: nVenn2: https://github.com/vqf/nVenn Web interface: https://vqf.github.io/nvenn2/nvenn2.html and https://degradome.uniovi.es/vqf/nvenn2/nvenn2.html Python interface: https://github.com/vqf/nVennPy. Also available from Pypi (https://pypi.org/project/nvenn2/ or from the command line with “pip install nvenn2”). R interface: https://github.com/vqf/nVennR2. Also available from CRAN (https://cran.r-project.org/package=nVennR2).
