# Supplementary material for: nVenn2: faster, simpler generalized quasi-proportional Venn diagrams
Source: Bioinform Adv. 2026 Jun 27;6(1):vbag183. doi: 10.1093/bioadv/vbag183 (PMC13353217; doi:10.1093/bioadv/vbag183)
Supplement: vbag183_Supplementary_Data [file vbag183_supplementary_data.zip › supplementary.pdf]

Supplementary Data

Pis-Vigil *et al.*

June 7, 2026

Supplementary Methods

Tangent class

Several methods use a class called *tangent* to store a slope without ambiguities. The object is created with two values, representing a Δx and a Δy or, equivalently, two points p_1 and p_2, where $\Delta x = x_{p_2} - x_{p_1}$ and $\Delta y = y_{p_2} - y_{p_1}$. Both values are stored, as well as a *quadrant* and a *slope* calculated from those values:

If $(\Delta x < 0 \wedge \Delta y \leq 0)$: $Quadrant = 1$; $Slope = \frac{\Delta y}{\Delta x}$

If $(\Delta x \geq 0 \wedge \Delta y < 0)$: $Quadrant = 2$; $Slope = -\frac{\Delta x}{\Delta y}$

If $(\Delta x > 0 \wedge \Delta y \geq 0)$: $Quadrant = 3$; $Slope = \frac{\Delta y}{\Delta x}$

If $(\Delta x \leq 0 \wedge \Delta y > 0)$: $Quadrant = 4$; $Slope = -\frac{\Delta x}{\Delta y}$

This ensures that each angle has a unique quadrant/slope combination and that every value is finite. In addition, angles do not need to be computed with expensive arctan operations. Another useful feature is that this class establishes a quick comparison between objects, where higher values mean *more counter-clockwise* starting at the negative x axis. Thus, an object with a higher quadrant is higher than an object with a lower quadrant. If two objects belong to the same quadrant, the object with a larger slope is higher.

Another useful operation is subtraction. Let t_1 and t_2 be two tangent objects. The operation $t_2 - t_1$ returns a tangent object equivalent to t_2 after rotating both objects so that t_1 sits at quadrant 1 with a slope of zero.

Adding set lines

The algorithm to add topologically correct set lines to a grid of circles representing regions runs in three steps.

Adding outer lines

For each set, the procedure iterates over all its regions and selects the leftmost circle (lowest x coordinate, ties do not matter). A reference tangent is created with quadrant 1 and slope 0.

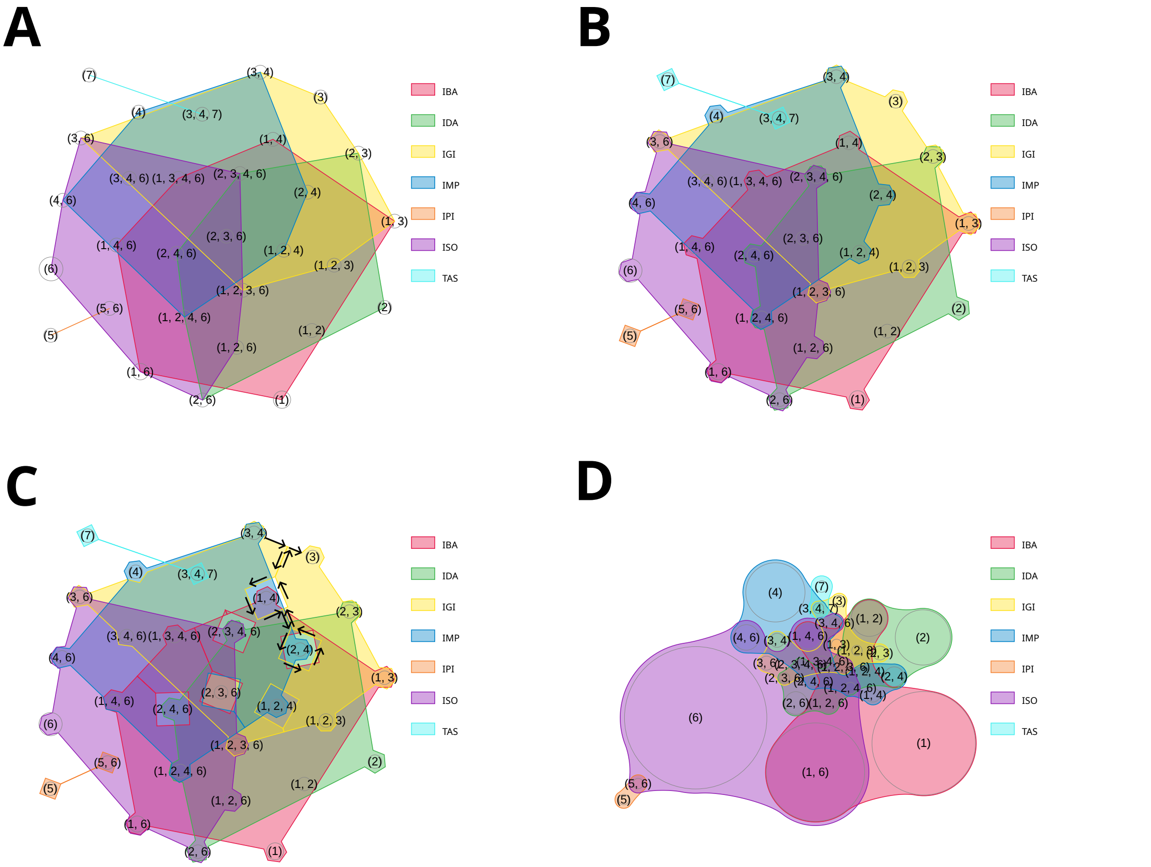

Supplementary Figure 1: Addition of set lines. (A) Addition of set lines that enclose all the regions in each set, and possibly regions not belonging to the set. (B) Addition of lines ensuring that the center of the circles defining the lines are inside the lines. (C) Correction of lines to exclude regions not belonging to each set. Arrows show one example where two regions are excluded from set 3 (IGI, yellow). (D) Final diagram. The universe for the figures contains genes included in the innate immune system GO category (GO: 0045087). Seven subsets with different GO evidence codes were generated: IBA (Inferred from Biological aspect of Ancestor), IDA (Inferred from Direct Assay), IGI (Inferred from Genetic Interaction), IMP (Inferred from Mutant Phenotype), IPI (Inferred from Physical Interaction), ISO (Inferred from Sequence Orthology) and TAS (Traceable Author Statement). Number lists in parentheses show the sets each region belongs to, in the same order as in the legend.

Then, the tangents from the center of this reference circle to the center of every other circle in the set are calculated, and the reference tangent is subtracted from each of them. The circle with the largest resulting tangent is then selected, and a segment is added from the center of the reference circle to the center of the selected circle. Then, the resulting tangent replaces the reference tangent, and the selected circle replaces the reference circle. This procedure is repeated until the

new selected circle is the first reference circle and the set line is closed (Supplementary Figure 1A).

Including centers

To ensure that the centers of each circle sit inside their sets, the intersections between the segments added in the previous step and the reference circles are calculated. Then, three segments that connect those intersections through the outside of the circle are added (Supplementary Figure 1B).

Fixing topology

The lines added in the previous step contain every circle in each set, but they may also contain circles that do not belong to that set. For each circle that does not belong to a set, the distance from its center to each of the set segments is calculated. If the closest point is not contained in the segment, the segment is discarded. The closest segment is selected, and split along the closest point. Six segments are added to the split to connect it again through the outside of the circle, which, after simulation, yields smooth set lines (Supplementary Figure 1C-D).

Steps

1 - Attract

Let the Venn diagram contain n_s sets. Each region in this diagram has a binary representation b_i with n_s bits expressing the sets it belongs and does not belong to. Each non-empty region is represented by a circle c_i with a radius r_i such that the area of the circle is proportional to the number of elements in this region. For each c_i and c_j circle pairs, relationships $f_{i,j}$ are calculated as the number of bit positions where both b_i and b_j contain a value of 1.

Briefly, circles representing regions are pseudorandomly placed on a lattice. Then a simulation is executed where circles are subjected to forces depending on their relationships and distance. For two circles c_i and c_j with a relationship of $f_{i,j}$ and radii r_i and r_j at a distance of $d_{i,j}$,

$$F_{i,j} = -k{f_{i,j}}^2\,(d_{i,j} - r_i - r_j) + R$$

The constant parameters k and R have been chosen to minimize the number of simulation cycles needed to finish this step. When updating the position of each circle c_i, a second friction force $F_f = -k_2 v_i$, is added, where v_i is the speed of c_i and k_2 is a constant parameter.

The simulation is run until $\max(v_i)$ is lower than a pre-defined value.

2 - Disperse

This step is very similar to step 1, but parameters $f_{i,j}$ are set to 1 for every pair and the value of R is much higher. As a result, all the forces are repulsive. The simulation is run until $\min(d_i) > \max(r_i)$.

3 - Minimize Compactness

Compactness (C) is defined as:

$$C = \sum_{i=0}^{n_r-1} \sum_{j=i+1}^{n_r} (\ln(f_{i,j}) + 2\ln(d_{i,j}))$$

Here, n_r is the number of non-empty regions in the Venn diagram. After calculating the first reference value of C, the algorithm iterates over each circle pair. At each iteration, the positions of both circles are swapped and C is calculated again. If the value after the exchange is lower than before, the swap is conserved and the reference is set to the new C value. If not, the swap is undone. The procedure is repeated indefinitely, cycling over the pairs multiple times if necessary, until the reference value has not changed for n_r consecutive iterations.

4 - Minimize Crossings

The procedure is the same as in step 3, but the number of line crossings (X) is used instead of compactness. To count crossings, each segment $p_1 \to p_2$ of a set line is compared to every segment $p_3 \to p_4$ from every other set line. First, a fast bounding box check filters segments that trivially do not cross. Then, for those comparisons where the bounding boxes of the segments overlap, four tangent objects are created:

$$
\begin{aligned}
t_1 &= tangent(p_1, p_3) \\
t_2 &= tangent(p_3, p_2) \\
t_3 &= tangent(p_2, p_4) \\
t_4 &= tangent(p_4, p_1)
\end{aligned}
$$

Both segments are considered as crossing if $((t_2 \leq t_3) \wedge (t_3 \leq t_4))$ or if $((t_3 \leq t_2) \wedge (t_4 \leq t_3))$. With this strategy, it is relatively simple to deal with special cases, such as two points with identical coordinates or segments that intersect at one of the segment points.

5 - Contract Lines

A simulation is built where each segment in each set line becomes a damped spring which sets a force for both segment points with magnitude $F = -kd + bv$, where k and b are constants, d is the distance between the points and v is the speed of the point. Contacts between the segments and the circles representing regions are also calculated.

In this setting, the diagram gets compacted as set lines shrink. Since the simulation depends on a time span in which motions are assumed linear, there are situations in which contacts will not be computed, as during that time span a segment may completely pass through a circle. For this reason, every 50 simulation cycles the topology of the diagram is assessed.

To calculate whether a circle is inside a set line, a horizontal segment is considered from the center of the circle to a point outside the bounding box of the set. This segment is compared to each segment of the set line to find crossings. If the number of segment crossings is odd, the circle is inside the set line. If a circle occupies an incorrect space, the simulation is returned to a previous topologically correct state, the time span is halved and the simulation continues. The correct state may be the initial state if necessary. If the topology of the diagram is correct, the current state substitutes the previous topologically correct state.

The step is finished when the 100-cycle average compactness is higher than the previous value and the compactness does not improve during 50 cycles. The state with the best compactness is saved and used for the following step.

6 - Compact regions

This step is very similar to the previous one, the difference being that a force between circles decaying with the inverse of the distance is added. This force further compacts the circles. The step is finished under the same conditions as step 5.

7 - Smooth set lines

For this step, circles are kept fixed at the positions at the end of step 6. The number of segments in set lines is increased by interpolation and a simulation is set with a damped spring-like force between segment points and an inverse-distance force between segment points and circles. This wraps lines more tightly around circles and makes the areas in the sets more similar to the areas in the regions.

The step is finished when the sum of the areas of the sets has not decreased for 50 cycles.

Supplementary Tables

Supplementary Table 1: Proteins in GO: 0045087 tagged with different evidence codes.

IBA	IDA	IGI	IMP	IPI	ISO	TAS
MGI:3041173	MGI:102676	MGI:1913963	MGI:1889575	MGI:3649078	MGI:2449119	MGI:105975
MGI:103206	MGI:102676	MGI:1921367	MGI:1333879	MGI:3774845	MGI:106388	MGI:97243
MGI:1333882	MGI:87906	MGI:105975	MGI:1913869		MGI:1333882	MGI:97244
MGI:2686159	MGI:109384	MGI:88385	MGI:1913869		MGI:2686159	
MGI:1889364	MGI:1098259	MGI:88385	MGI:1277186		MGI:104729	
MGI:1918731	MGI:101773	MGI:2135589	MGI:1921494		MGI:87986	
MGI:1918731	MGI:2687214	MGI:88489	MGI:2138446		MGI:1918731	
MGI:88022	MGI:2687214	MGI:107539	MGI:1261775		MGI:1918731	
MGI:104984	MGI:106211	MGI:1914320	MGI:2685628		MGI:88022	
MGI:1201793	MGI:1929744	MGI:1914320	MGI:1934368		MGI:104984	
MGI:2656551	MGI:1929760	MGI:2444773	MGI:1934368		MGI:1201793	
MGI:3528599	MGI:108445	MGI:96923	MGI:3039602		MGI:2656551	
MGI:3528602	MGI:2135589	MGI:96924	MGI:3039602		MGI:3528599	
MGI:1921733	MGI:2135589	MGI:108005	MGI:2687214		MGI:3528602	
MGI:1932101	MGI:1861431	MGI:1328352	MGI:2687214		MGI:1932101	
MGI:98229	MGI:97838	MGI:1328352	MGI:105975		MGI:1932101	
MGI:3630303	MGI:1915168	MGI:1328352	MGI:105975		MGI:1932101	
MGI:3045315	MGI:1194884	MGI:1328352	MGI:87931		MGI:101919	
MGI:1338036	MGI:1340905	MGI:1349420	MGI:88385		MGI:101919	
MGI:1914385	MGI:2446089	MGI:1349420	MGI:97545		MGI:98229	
MGI:2137431	MGI:102772	MGI:1349420	MGI:1298389		MGI:98229	
MGI:2137431	MGI:95666	MGI:1349420	MGI:1861232		MGI:88051	
MGI:2177191	MGI:1926263	MGI:1349420	MGI:1278313		MGI:1919148	
MGI:1916800	MGI:97072	MGI:1349420	MGI:1927070		MGI:3036238	
MGI:1917562	MGI:2429943	MGI:3622649	MGI:1927070		MGI:3649094	
MGI:1889272	MGI:2140937	MGI:3622649	MGI:1919349		MGI:88059	
MGI:108443	MGI:2444421	MGI:3622649	MGI:1918673		MGI:88059	
MGI:88318	MGI:1923324	MGI:3622649	MGI:1918673		MGI:88059	
MGI:109294	MGI:3605620	MGI:3622649	MGI:1352467		MGI:107387	
MGI:2443411	MGI:4359647	MGI:3622649	MGI:1918788		MGI:88070	
MGI:3032626	MGI:3641753	MGI:1346060	MGI:95496		MGI:1914416	
MGI:2153249	MGI:1933113	MGI:3045213	MGI:107179		MGI:99474	
MGI:2387602	MGI:95851	MGI:109274	MGI:102858		MGI:107168	
MGI:2442261	MGI:95936	MGI:98923	MGI:1918876		MGI:107168	
MGI:3036291	MGI:95938	MGI:98923	MGI:1913534		MGI:88127	
MGI:1349412	MGI:95945	MGI:2143362	MGI:3641753		MGI:1346330	
MGI:1920399	MGI:1306817		MGI:1925558		MGI:1346330	
MGI:3624119	MGI:1347080		MGI:95915		MGI:1337994	
MGI:1917060	MGI:99449		MGI:1915183		MGI:1891828	
MGI:3588267	MGI:1915963		MGI:1915183		MGI:1338036	

IBA	IDA	IGI	IMP	IPI	ISO	TAS
MGI:1298389	MGI:1913391		MGI:105112		MGI:1914385	
MGI:1861232	MGI:107668		MGI:105112		MGI:2137431	
MGI:1861231	MGI:107664		MGI:105112		MGI:2137431	
MGI:1345151	MGI:107659		MGI:1918836		MGI:2389572	
MGI:1861431	MGI:2450574		MGI:1918836		MGI:3647286	
MGI:88512	MGI:96540		MGI:1338888		MGI:1916800	
MGI:1859822	MGI:107936		MGI:2152414		MGI:88216	
MGI:94880	MGI:1890474		MGI:1859179		MGI:1194505	
MGI:94882	MGI:1890474		MGI:1096873		MGI:1194505	
MGI:94883	MGI:1890474		MGI:1859212		MGI:1917562	
MGI:99584	MGI:1890474		MGI:1100508		MGI:1917562	
MGI:99583	MGI:107420		MGI:2145579		MGI:1889272	
MGI:99582	MGI:107420		MGI:2662547		MGI:1889272	
MGI:99581	MGI:107567		MGI:1196250		MGI:1343177	
MGI:99580	MGI:1100508		MGI:1196250		MGI:88251	
MGI:99579	MGI:1098268		MGI:106588		MGI:88256	
MGI:99591	MGI:1098240		MGI:106588		MGI:108443	
MGI:99590	MGI:107539		MGI:1925000		MGI:108443	
MGI:99588	MGI:107539		MGI:1915864		MGI:2685628	
MGI:99587	MGI:107539		MGI:3650490		MGI:96544	
MGI:99586	MGI:107539		MGI:96924		MGI:1312921	
MGI:99585	MGI:107538		MGI:1100846		MGI:102709	
MGI:1345152	MGI:107538		MGI:2676278		MGI:98262	
MGI:1915259	MGI:2662547		MGI:2676278		MGI:88320	
MGI:1913548	MGI:106588		MGI:108005		MGI:88318	
MGI:3639039	MGI:106588		MGI:108005		MGI:88318	
MGI:3630381	MGI:2140219		MGI:108005		MGI:107899	
MGI:3630383	MGI:1918518		MGI:107711		MGI:96617	
MGI:3630385	MGI:1918518		MGI:1351641		MGI:96534	
MGI:3630390	MGI:1914320		MGI:1343135		MGI:96534	
MGI:3642780	MGI:1330832		MGI:1931595		MGI:1860383	
MGI:3646688	MGI:96923		MGI:1931595		MGI:1860383	
MGI:94881	MGI:96924		MGI:2653833		MGI:1860383	
MGI:3808881	MGI:3718546		MGI:2141990		MGI:3039602	
MGI:102509	MGI:97009		MGI:2429611		MGI:3039602	
MGI:3709048	MGI:97142		MGI:2429611		MGI:109294	
MGI:3711900	MGI:106612		MGI:2429397		MGI:1926446	
MGI:3705236	MGI:2667185		MGI:2429397		MGI:2443411	
MGI:3709605	MGI:104741		MGI:2429397		MGI:2442359	
MGI:3611585	MGI:104741		MGI:2429397		MGI:2442359	
MGI:3708769	MGI:104741		MGI:2429397		MGI:109531	
MGI:3705230	MGI:1931595		MGI:103187		MGI:109531	
MGI:3645033	MGI:2653833		MGI:1914495		MGI:1347245	
MGI:99592	MGI:1928368		MGI:1914495		MGI:2158560	
MGI:102516	MGI:97361		MGI:1924963		MGI:88385	

IBA	IDA	IGI	IMP	IPI	ISO	TAS
MGI:102514	MGI:2443180		MGI:1924963		MGI:3646434	
MGI:1096878	MGI:2443180		MGI:1914005		MGI:2442261	
MGI:1338754	MGI:2442295		MGI:99523		MGI:2442261	
MGI:2179198	MGI:1098586		MGI:2443180		MGI:2442261	
MGI:2179205	MGI:894284		MGI:2443180		MGI:2442261	
MGI:2179197	MGI:102789		MGI:96055		MGI:2442261	
MGI:2672966	MGI:1917084		MGI:1861441		MGI:2442261	
MGI:2672972	MGI:109432		MGI:1341850		MGI:1333869	
MGI:2672974	MGI:1861032		MGI:1891456		MGI:99484	
MGI:2672976	MGI:1345275		MGI:1891456		MGI:108445	
MGI:3033850	MGI:893577		MGI:1891456		MGI:108445	
MGI:3033850	MGI:1890156		MGI:1922484		MGI:1298389	
MGI:1931560	MGI:1921982		MGI:1924983		MGI:1861232	
MGI:1931560	MGI:107363		MGI:1924983		MGI:1345151	
MGI:1931560	MGI:107370		MGI:1915252		MGI:1918433	
MGI:108021	MGI:107362		MGI:1328352		MGI:1100517	
MGI:108020	MGI:1342296		MGI:1328352		MGI:1100517	
MGI:1858237	MGI:98734		MGI:1349420		MGI:1298230	
MGI:1858238	MGI:96824		MGI:1349420		MGI:3606482	
MGI:1890463	MGI:96824		MGI:3622649		MGI:1918943	
MGI:1890464	MGI:96824		MGI:3622649		MGI:2152907	
MGI:3528616	MGI:96824		MGI:1927664		MGI:1345961	
MGI:1349469	MGI:96824		MGI:1927664		MGI:1098296	
MGI:1890169	MGI:109274		MGI:1921998		MGI:1933107	
MGI:3588218	MGI:1925027		MGI:1923724		MGI:88489	
MGI:109324	MGI:1321389		MGI:103036		MGI:88508	
MGI:95496	MGI:1858730		MGI:103036		MGI:1859822	
MGI:1340905	MGI:1336882		MGI:103035		MGI:1859822	
MGI:1341158	MGI:98932		MGI:103035		MGI:1891441	
MGI:102772	MGI:1917656		MGI:1921982		MGI:1891441	
MGI:95666	MGI:105059		MGI:1921982		MGI:1333815	
MGI:1926263	MGI:2143362		MGI:1921982		MGI:1932682	
MGI:97072	MGI:103072		MGI:1929658		MGI:1316658	
MGI:2429943			MGI:2147032		MGI:1316658	
MGI:2140937			MGI:2147032		MGI:88574	
MGI:2444421			MGI:2385852		MGI:2384959	
MGI:1923324			MGI:2152213		MGI:1921506	
MGI:3605620			MGI:1346060		MGI:1098274	
MGI:4359647			MGI:96824		MGI:1916851	
MGI:3643814			MGI:1913521		MGI:1916885	
MGI:3645205			MGI:2441706		MGI:1203520	
MGI:3652173			MGI:1328317		MGI:1197015	
MGI:3649299			MGI:1328317		MGI:1925022	
MGI:3701966			MGI:1328317		MGI:103064	
MGI:3701966			MGI:1328317		MGI:103064	

IBA	IDA	IGI	IMP	IPI	ISO	TAS
MGI:3701968			MGI:1328317		MGI:103064	
MGI:3701968			MGI:1328317		MGI:2384570	
MGI:3701970			MGI:2442815		MGI:2384570	
MGI:3701970			MGI:2442815		MGI:94880	
MGI:3701972			MGI:2442815		MGI:94882	
MGI:3701972			MGI:2442815		MGI:94883	
MGI:3701974			MGI:1917700		MGI:99583	
MGI:3701974			MGI:1859307		MGI:99582	
MGI:3649573			MGI:1922333		MGI:99581	
MGI:3649573			MGI:2441787		MGI:99580	
MGI:2442492			MGI:1927449		MGI:99579	
MGI:1925558					MGI:99591	
MGI:2676308					MGI:99590	
MGI:99448					MGI:99588	
MGI:1347080					MGI:99587	
MGI:96428					MGI:99586	
MGI:96429					MGI:99585	
MGI:101847					MGI:1345152	
MGI:3646410					MGI:3630381	
MGI:2138302					MGI:3630383	
MGI:2442822					MGI:3630385	
MGI:2138243					MGI:3630390	
MGI:3041120					MGI:3642780	
MGI:3695276					MGI:3646688	
MGI:3584522					MGI:3808881	
MGI:1918836					MGI:102509	
MGI:1918836					MGI:3709048	
MGI:1915963					MGI:3708769	
MGI:1915963					MGI:1096878	
MGI:1915963					MGI:2385955	
MGI:1933382					MGI:2385955	
MGI:1933382					MGI:2684967	
MGI:1933382					MGI:3033850	
MGI:1913391					MGI:3033850	
MGI:1913391					MGI:1099786	
MGI:1913391					MGI:1916442	
MGI:2686976					MGI:2445102	
MGI:2686976					MGI:1931560	
MGI:2686976					MGI:1931560	
MGI:1921732					MGI:1931560	
MGI:1921732					MGI:1931560	
MGI:1921732					MGI:1931560	
MGI:107668					MGI:1933786	
MGI:107668					MGI:1933786	
MGI:107666					MGI:94919	

IBA	IDA	IGI	IMP	IPI	ISO	TAS
MGI:107666					MGI:2672905	
MGI:107664					MGI:1913619	
MGI:107664					MGI:1349469	
MGI:107663					MGI:1353449	
MGI:107663					MGI:1914440	
MGI:107662					MGI:1918673	
MGI:107662					MGI:97838	
MGI:107661					MGI:1890169	
MGI:107661					MGI:101910	
MGI:107659					MGI:101910	
MGI:107659					MGI:101910	
MGI:109210					MGI:101910	
MGI:109210					MGI:109324	
MGI:2676324					MGI:1913544	
MGI:2676324					MGI:102547	
MGI:2667155					MGI:1919429	
MGI:2667155					MGI:2441731	
MGI:3641425					MGI:1100500	
MGI:3641425					MGI:1278319	
MGI:3649418					MGI:95640	
MGI:3649418					MGI:3782011	
MGI:3649260					MGI:3708786	
MGI:3649260					MGI:2429943	
MGI:1097683					MGI:95675	
MGI:1097683					MGI:99846	
MGI:107658					MGI:107757	
MGI:1098243					MGI:103170	
MGI:107657					MGI:2138584	
MGI:107657					MGI:1932723	
MGI:2667156					MGI:1932723	
MGI:2667156					MGI:2442232	
MGI:2683287					MGI:2442232	
MGI:2683287					MGI:2442492	
MGI:3647279					MGI:95805	
MGI:2450574					MGI:95832	
MGI:2448469					MGI:95851	
MGI:2448469					MGI:109267	
MGI:107729					MGI:109256	
MGI:1926259					MGI:2448415	
MGI:3644953					MGI:3649078	
MGI:1929612					MGI:3649078	
MGI:1890474					MGI:1344360	
MGI:107420					MGI:3036234	
MGI:107420					MGI:2385923	
MGI:107420					MGI:96113	

IBA	IDA	IGI	IMP	IPI	ISO	TAS
MGI:107567					MGI:96244	
MGI:1926262					MGI:99517	
MGI:96877					MGI:105384	
MGI:107540					MGI:96242	
MGI:107539					MGI:1917360	
MGI:107538					MGI:1917360	
MGI:2442965					MGI:2138302	
MGI:1336161					MGI:2138302	
MGI:1336161					MGI:2138302	
MGI:1336162					MGI:1918836	
MGI:1336162					MGI:99449	
MGI:1929720					MGI:1101055	
MGI:1929720					MGI:3698419	
MGI:1196275					MGI:1913391	
MGI:1196275					MGI:1913391	
MGI:2662547					MGI:2686976	
MGI:2662547					MGI:2686976	
MGI:2685002					MGI:1921732	
MGI:3530275					MGI:1921732	
MGI:3530275					MGI:107658	
MGI:2443965					MGI:1098243	
MGI:2443965					MGI:107657	
MGI:1196250					MGI:107657	
MGI:106588					MGI:107656	
MGI:1098776					MGI:107656	
MGI:2445060					MGI:107655	
MGI:2140219					MGI:107655	
MGI:3045299					MGI:107654	
MGI:1321404					MGI:96434	
MGI:1341909					MGI:96448	
MGI:2445027					MGI:107729	
MGI:109298					MGI:107729	
MGI:2143163					MGI:107729	
MGI:88492					MGI:107729	
MGI:1330832					MGI:107729	
MGI:1859396					MGI:1929612	
MGI:1098644					MGI:96539	
MGI:3780953					MGI:96539	
MGI:1913539					MGI:96540	
MGI:3779286					MGI:96540	
MGI:108005					MGI:104579	
MGI:108005					MGI:1890474	
MGI:97822					MGI:2181693	
MGI:1890645					MGI:1921579	
MGI:1340031					MGI:1921579	

IBA	IDA	IGI	IMP	IPI	ISO	TAS
MGI:3612191					MGI:1921579	
MGI:2684861					MGI:96572	
MGI:3582959					MGI:96573	
MGI:2180860					MGI:1917822	
MGI:2180860					MGI:107420	
MGI:97430					MGI:107420	
MGI:97430					MGI:2429603	
MGI:2149633					MGI:96590	
MGI:2149633					MGI:96591	
MGI:2140770					MGI:1859179	
MGI:2140770					MGI:1859179	
MGI:2180856					MGI:1859179	
MGI:2180856					MGI:1859179	
MGI:2180855					MGI:1859212	
MGI:2180855					MGI:96395	
MGI:97429					MGI:107567	
MGI:97429					MGI:107567	
MGI:2180853					MGI:107567	
MGI:2180853					MGI:107567	
MGI:2180852					MGI:107567	
MGI:2180852					MGI:1926262	
MGI:2180850					MGI:1926262	
MGI:2180850					MGI:1926262	
MGI:2180849					MGI:1926262	
MGI:2180849					MGI:1926262	
MGI:1344390					MGI:1855694	
MGI:1344390					MGI:1855694	
MGI:1098801					MGI:1855694	
MGI:3577015					MGI:1202301	
MGI:1933117					MGI:96628	
MGI:1919489					MGI:96628	
MGI:2144613					MGI:96629	
MGI:104662					MGI:96493	
MGI:107682					MGI:1932051	
MGI:1914005					MGI:1098268	
MGI:107170					MGI:1196275	
MGI:104641					MGI:1196275	
MGI:107741					MGI:1196275	
MGI:1931465					MGI:1196275	
MGI:1918910					MGI:2662547	
MGI:97897					MGI:1196250	
MGI:103290					MGI:96698	
MGI:103289					MGI:1918039	
MGI:2149590					MGI:2445077	
MGI:2442858					MGI:2445077	

IBA	IDA	IGI	IMP	IPI	ISO	TAS
MGI:2442858					MGI:96745	
MGI:1891456					MGI:96745	
MGI:1890465					MGI:96748	
MGI:1858598					MGI:1098776	
MGI:1925666					MGI:1098776	
MGI:2156378					MGI:104663	
MGI:1919206					MGI:2445060	
MGI:1913416					MGI:1921738	
MGI:1927468					MGI:96837	
MGI:1921386					MGI:96837	
MGI:2441788					MGI:96837	
MGI:109297					MGI:106651	
MGI:1913770					MGI:1341909	
MGI:103063					MGI:107470	
MGI:103039					MGI:2385115	
MGI:1919762					MGI:107448	
MGI:1919762					MGI:1346877	
MGI:1929658					MGI:1346877	
MGI:98734					MGI:1346861	
MGI:3710083					MGI:1920955	
MGI:2147032					MGI:88492	
MGI:3040056					MGI:1330832	
MGI:2182965					MGI:1298379	
MGI:2182965					MGI:2444773	
MGI:2152213					MGI:2444773	
MGI:1341295					MGI:2444773	
MGI:1346060					MGI:2444773	
MGI:2156367					MGI:2444773	
MGI:96824					MGI:2444773	
MGI:96824					MGI:2444773	
MGI:1858171					MGI:96923	
MGI:1341296					MGI:96924	
MGI:2176882					MGI:96924	
MGI:2176882					MGI:96924	
MGI:2176887					MGI:96924	
MGI:1932389					MGI:96924	
MGI:3045226					MGI:1859396	
MGI:3045221					MGI:1859396	
MGI:3045213					MGI:1927165	
MGI:2386643					MGI:1098644	
MGI:2386643					MGI:1098644	
MGI:2441706					MGI:1098644	
MGI:108072					MGI:1098644	
MGI:1923551					MGI:1344333	
MGI:1913150					MGI:1344333	

IBA	IDA	IGI	IMP	IPI	ISO	TAS
MGI:1923239					MGI:2136841	
MGI:1914123					MGI:1100512	
MGI:3646853					MGI:1915600	
MGI:2137352					MGI:1915600	
MGI:2137353					MGI:108005	
MGI:1338757					MGI:108005	
MGI:2137355					MGI:108005	
MGI:1923931					MGI:108005	
MGI:4821183					MGI:2442218	
MGI:1913847					MGI:1860418	
MGI:1921985					MGI:1351625	
MGI:1916347					MGI:97283	
MGI:1861440					MGI:109601	
MGI:106657					MGI:2149332	
MGI:1337056					MGI:97822	
MGI:97904					MGI:97822	
MGI:109274					MGI:97822	
MGI:98178					MGI:97297	
MGI:4821256					MGI:1890645	
MGI:4821257					MGI:97312	
MGI:3035181					MGI:104741	
MGI:2385051					MGI:1340031	
MGI:2137359					MGI:2444070	
MGI:4821264					MGI:3612191	
MGI:1914104					MGI:3612191	
MGI:2684869					MGI:3612191	
MGI:1890659					MGI:3612191	
MGI:2684881					MGI:2684861	
MGI:2384814					MGI:2684861	
MGI:3645218					MGI:3582959	
MGI:3648996					MGI:3582959	
MGI:3647365					MGI:2653833	
MGI:1931835					MGI:2443697	
MGI:2664992					MGI:3056570	
MGI:3045276					MGI:1890518	
MGI:1889623					MGI:3056600	
MGI:3036269					MGI:2145528	
MGI:2685298					MGI:2141990	
MGI:2685298					MGI:2141990	
MGI:2684862					MGI:2444084	
MGI:1914199					MGI:1100525	
MGI:2387430					MGI:1928368	
MGI:2387432					MGI:1341839	
MGI:1914775					MGI:1341839	
MGI:2447992					MGI:2429397	

IBA	IDA	IGI	IMP	IPI	ISO	TAS
MGI:2142077					MGI:2429397	
MGI:1918178					MGI:2429397	
MGI:3612190					MGI:2429397	
MGI:2685640					MGI:2429397	
MGI:2687279					MGI:1855692	
MGI:3642989					MGI:2154441	
MGI:1349462					MGI:2679787	
MGI:1344410					MGI:97374	
MGI:1859307					MGI:2444210	
MGI:1859307					MGI:1352462	
MGI:1859307					MGI:2180860	
MGI:2679720					MGI:2180860	
MGI:98943					MGI:2180860	
MGI:1916618					MGI:2180860	
MGI:1916618					MGI:2180860	
MGI:1914951					MGI:97430	
MGI:1923897					MGI:97430	
MGI:2384800					MGI:97430	
MGI:3652032					MGI:97430	
MGI:3616889					MGI:97430	
MGI:3651686					MGI:2149633	
MGI:2183434					MGI:2149633	
MGI:3582777					MGI:2149633	
MGI:1915471					MGI:2149633	
MGI:2445041					MGI:2149633	
MGI:2670994					MGI:2140770	
MGI:3649773					MGI:2140770	
MGI:107506					MGI:2140770	
MGI:1913357					MGI:2140770	
MGI:1927449					MGI:2140770	
MGI:1915167					MGI:2180856	
MGI:1915167					MGI:2180856	
MGI:1918046					MGI:2180856	
MGI:1923573					MGI:2180856	
MGI:1918414					MGI:2180856	
					MGI:2180855	
					MGI:2180855	
					MGI:2180855	
					MGI:2180855	
					MGI:2180855	
					MGI:97429	
					MGI:97429	
					MGI:97429	
					MGI:97429	
					MGI:97429	

IBA	IDA	IGI	IMP	IPI	ISO	TAS
					MGI:2180853	
					MGI:2180853	
					MGI:2180853	
					MGI:2180853	
					MGI:2180853	
					MGI:2180850	
					MGI:2180850	
					MGI:2180850	
					MGI:2180849	
					MGI:1098801	
					MGI:3577015	
					MGI:3577015	
					MGI:1340806	
					MGI:1340806	
					MGI:1933117	
					MGI:1933117	
					MGI:1919489	
					MGI:108202	
					MGI:2443226	
					MGI:2443226	
					MGI:97572	
					MGI:97572	
					MGI:102520	
					MGI:102520	
					MGI:97583	
					MGI:97583	
					MGI:2144613	
					MGI:97584	
					MGI:97584	
					MGI:2159342	
					MGI:2159342	
					MGI:101842	
					MGI:97616	
					MGI:1916832	
					MGI:893575	
					MGI:104662	
					MGI:2681836	
					MGI:1917678	
					MGI:1921664	
					MGI:1914315	
					MGI:1924086	
					MGI:1914736	
					MGI:97747	
					MGI:1915107	
					MGI:1298204	

IBA	IDA	IGI	IMP	IPI	ISO	TAS
					MGI:1859638	
					MGI:104779	
					MGI:104779	
					MGI:1344375	
					MGI:1913895	
					MGI:97806	
					MGI:97806	
					MGI:107170	
					MGI:107170	
					MGI:107170	
					MGI:107170	
					MGI:97815	
					MGI:104641	
					MGI:104641	
					MGI:1931749	
					MGI:1931751	
					MGI:107741	
					MGI:107741	
					MGI:107741	
					MGI:1931465	
					MGI:1931465	
					MGI:1922248	
					MGI:109292	
					MGI:109431	
					MGI:109431	
					MGI:1918910	
					MGI:1918910	
					MGI:1314635	
					MGI:2384294	
					MGI:103290	
					MGI:103290	
					MGI:103290	
					MGI:1923688	
					MGI:2442858	
					MGI:2442858	
					MGI:2442858	
					MGI:2442858	
					MGI:1914128	
					MGI:1914128	
					MGI:1891456	
					MGI:1891456	
					MGI:1891456	
					MGI:1891456	
					MGI:1925666	
					MGI:2153340	

IBA	IDA	IGI	IMP	IPI	ISO	TAS
					MGI:1915095	
					MGI:1914914	
					MGI:1919206	
					MGI:1919206	
					MGI:1919206	
					MGI:1344401	
					MGI:1924983	
					MGI:1922078	
					MGI:1922078	
					MGI:1351455	
					MGI:1914498	
					MGI:1333780	
					MGI:1333780	
					MGI:1913416	
					MGI:1927468	
					MGI:1927468	
					MGI:1921386	
					MGI:1349457	
					MGI:2444223	
					MGI:106603	
					MGI:894668	
					MGI:894669	
					MGI:894667	
					MGI:894672	
					MGI:894671	
					MGI:1919260	
					MGI:3709608	
					MGI:894696	
					MGI:1918177	
					MGI:1891831	
					MGI:1918764	
					MGI:2441788	
					MGI:2441788	
					MGI:2441788	
					MGI:1277989	
					MGI:107157	
					MGI:107157	
					MGI:108563	
					MGI:1351663	
					MGI:1353620	
					MGI:1890457	
					MGI:2140796	
					MGI:2140796	
					MGI:2140796	
					MGI:2140796	

IBA	IDA	IGI	IMP	IPI	ISO	TAS
					MGI:103182	
					MGI:1329012	
					MGI:1929481	
					MGI:2159728	
					MGI:1353616	
					MGI:103241	
					MGI:1935129	
					MGI:1913770	
					MGI:98325	
					MGI:1277151	
					MGI:1860188	
					MGI:109561	
					MGI:109561	
					MGI:107931	
					MGI:98397	
					MGI:103063	
					MGI:103063	
					MGI:103063	
					MGI:103063	
					MGI:103039	
					MGI:103039	
					MGI:1919762	
					MGI:1919762	
					MGI:1919762	
					MGI:1919762	
					MGI:1919762	
					MGI:1919762	
					MGI:1919762	
					MGI:2138351	
					MGI:1891690	
					MGI:1913763	
					MGI:98474	
					MGI:98483	
					MGI:98484	
					MGI:103027	
					MGI:1918648	
					MGI:1918648	
					MGI:1918648	
					MGI:1289308	
					MGI:1289308	
					MGI:1929658	
					MGI:1929658	
					MGI:1929658	
					MGI:1929658	
					MGI:1929658	

IBA	IDA	IGI	IMP	IPI	ISO	TAS
					MGI:103270	
					MGI:98725	
					MGI:2147032	
					MGI:2147032	
					MGI:2147032	
					MGI:2147032	
					MGI:3040056	
					MGI:3040056	
					MGI:3040056	
					MGI:3040056	
					MGI:2182965	
					MGI:2182965	
					MGI:2152213	
					MGI:2152213	
					MGI:2152213	
					MGI:2152213	
					MGI:2152213	
					MGI:2385084	
					MGI:2385084	
					MGI:1341295	
					MGI:1341295	
					MGI:1341295	
					MGI:1346060	
					MGI:1346060	
					MGI:1346060	
					MGI:1346060	
					MGI:1346060	
					MGI:2156367	
					MGI:2156367	
					MGI:2156367	
					MGI:2156367	
					MGI:96824	
					MGI:96824	
					MGI:96824	
					MGI:96824	
					MGI:96824	
					MGI:96824	
					MGI:1858171	
					MGI:1341296	
					MGI:1341296	
					MGI:1341296	
					MGI:1341296	
					MGI:2176882	
					MGI:2176887	
					MGI:2176887	

IBA	IDA	IGI	IMP	IPI	ISO	TAS
					MGI:1932389	
					MGI:1932389	
					MGI:1932389	
					MGI:1932389	
					MGI:104798	
					MGI:104798	
					MGI:1196377	
					MGI:3041165	
					MGI:3041165	
					MGI:106295	
					MGI:101835	
					MGI:108041	
					MGI:2441706	
					MGI:108072	
					MGI:108072	
					MGI:108072	
					MGI:1913150	
					MGI:1923239	
					MGI:1328317	
					MGI:1328317	
					MGI:1914123	
					MGI:1860040	
					MGI:3646853	
					MGI:3646853	
					MGI:3646853	
					MGI:2137352	
					MGI:2137352	
					MGI:2137353	
					MGI:1933302	
					MGI:2137355	
					MGI:2137355	
					MGI:2137355	
					MGI:4821183	
					MGI:4821183	
					MGI:4821183	
					MGI:1913847	
					MGI:1921985	
					MGI:1916347	
					MGI:1916347	
					MGI:106657	
					MGI:106657	
					MGI:106657	
					MGI:102749	
					MGI:102749	
					MGI:102749	

IBA	IDA	IGI	IMP	IPI	ISO	TAS
					MGI:1337056	
					MGI:1337056	
					MGI:97904	
					MGI:109274	
					MGI:98178	
					MGI:98178	
					MGI:98178	
					MGI:4821257	
					MGI:4821257	
					MGI:4821257	
					MGI:3035181	
					MGI:3035181	
					MGI:3035181	
					MGI:2385051	
					MGI:2385051	
					MGI:2385051	
					MGI:2385051	
					MGI:1917057	
					MGI:1917057	
					MGI:1914104	
					MGI:2384814	
					MGI:2685298	
					MGI:2685298	
					MGI:1914775	
					MGI:1914775	
					MGI:1926264	
					MGI:1919604	
					MGI:3039573	
					MGI:102960	
					MGI:1929470	
					MGI:1929470	
					MGI:1277211	
					MGI:1277211	
					MGI:1334459	
					MGI:1349462	
					MGI:1858216	
					MGI:1914500	
					MGI:1914049	
					MGI:109353	
					MGI:3588256	
					MGI:102674	
					MGI:1859307	
					MGI:1859307	
					MGI:1859307	
					MGI:101857	

IBA	IDA	IGI	IMP	IPI	ISO	TAS
					MGI:101857	
					MGI:1921520	
					MGI:1859645	
					MGI:1922091	
					MGI:3045318	
					MGI:1922333	
					MGI:1096399	
					MGI:98923	
					MGI:98933	
					MGI:2679720	
					MGI:1916618	
					MGI:1916618	
					MGI:98958	
					MGI:104517	
					MGI:95606	
					MGI:2444233	
					MGI:894689	
					MGI:894689	
					MGI:109484	
					MGI:1927449	
					MGI:1926031	
					MGI:1915167	
					MGI:1915167	
					MGI:1918046	
					MGI:1926134	
					MGI:1920131	
					MGI:1923573	
					MGI:1923573	
					MGI:2444393	
					MGI:1913470	
					MGI:3527792	
					MGI:3527792	
					MGI:2177308	
					MGI:1341258	
